# Exposure to dogs and cats and risk of asthma: A retrospective study

**DOI:** 10.1371/journal.pone.0282184

**Published:** 2023-03-08

**Authors:** Yu Taniguchi, Maasa Kobayashi

**Affiliations:** 1 Japan Environment and Children’s Study Programme Office, National Institute for Environmental Studies, Ibaraki, Japan; 2 Research Team for Social Participation and Community Health, Tokyo Metropolitan Institute of Gerontology, Tokyo, Japan; 3 Graduate School of Nursing Science, St. Luke’s International University, Tokyo, Japan; Kyung Hee University School of Medicine, REPUBLIC OF KOREA

## Abstract

Findings on the relationship between pet exposure and asthma in western countries are inconsistent. This retrospective study examined the association of owning a dog or cat with the onset of asthma in Japanese people. We also investigated whether there is a critical window during which exposure to dogs and cats can reduce the risk of asthma by stratifying the analysis by the age at which pet ownership began. We analyzed data collected in an internet survey conducted by the Japan Pet Food Association in 2021. Valid data were obtained from 4290 participants for analysis of dog ownership and 4308 participants for analysis of cat ownership. In these respective groups, 41.2% had owned a dog and 26.5% had owned a cat. During the follow-up period, 5.7% of dog owners and 14.8% of non-dog owners developed asthma, as did 5.6% of cat owners and 13.5% of non-cat owners. On binomial logistic regression analysis, participants who had not owned a dog had an odds ratio (OR) of 2.01 (95% confidence interval (CI): 1.45–2.78) of developing asthma compared to those who had owned a dog after adjustment for sociodemographic characteristics. The corresponding OR of asthma onset among participants who had not owned a cat was 2.24 (95%CI: 1.56–3.23). Stratified analysis showed that while younger participants who had not owned a dog had higher ORs of developing asthma, those who had not owned a cat had similar ORs of asthma onset across all age categories. These results suggest that while there may be a critical window in early life during which exposure to dogs can prevent asthma onset, the protective effect of cat exposure is constant across all ages in Japan.

## Introduction

A variety of studies have demonstrated the psychological, physiological, and social benefits of human–animal interaction. Owning a pet such as a dog or cat has a positive effect on physical activity level [1] and prosocial and humane behavior [2] among children, and affects blood pressure [3], hyperlipidemia [3], physical activity [3–5], obesity [3], autonomic function [3], survival rate [6], social capital and civic engagement [7], and social contact and social isolation [5] among adults and/or the elderly.

However, pet ownership also has negative effects. Exposure to furry pets is a potential risk factor for allergic diseases such as asthma, because they harbor animal-derived allergens, which are classified as a risk and aggravating factor for asthma in the Asthma Prevention and Management Guidelines 2021. Several studies have reported that owning a pet has a negative effect on childhood asthma [8, 9]. In fact, clinicians may discourage pet ownership by families with children who develop asthma in infancy to improve the child’s asthma symptoms. On the other hand, there is also evidence that pet exposure can have a protective effect on the development of childhood asthma [10, 11]. Although a systematic review of 32 studies suggested that pet exposure was associated with a slight increase in the risk of asthma and wheezing in older children [12], another meta-analysis of 32 studies reported that cat exposure had a slight preventive effect on asthma, while exposure to dogs slightly increased the risk of asthma [13]. Given these inconsistent findings in western countries, the relationship between pet ownership and the onset of asthma may differ depending on the characteristics of the study population, such as the environment in which animals are kept (indoors or outdoors) and the period of pet exposure.

We previously reported associations of pet ownership with wheezing and asthma among 410 Japanese children [14]. Our results showed that long-term and toddler-age dog and/or cat owners had consistently lower risks of wheezing and asthma until age 6 years compared with never owners, suggesting that early postnatal exposure to pets in modern Japan may have a protective effect on the onset of asthma. This study had a relatively small sample size and focused on young participants, however, and the association of dog and/or cat ownership with asthma in Japan requires further study.

A recent study of over 1000 7- to 8-year-old children reported that the prevalence of allergic diseases decreases in a dose-dependent manner with the number of pets living with the child during their first year of life, suggesting that owning dogs and/or cats could protect against allergy development [15]. Whether owning a dog or cat has a protective effect against asthma in Japanese populations remains unknown. To our knowledge, no study has examined the critical window during which dog or cat ownership protects against the development of asthma.

This retrospective study had two main objectives: (ⅰ) to examine the association of owning a dog or cat with the onset of asthma in Japanese people across a wide range of ages from birth to old age; and (ⅱ) to identify the critical window during which dog or cat ownership reduces the risk of asthma by performing stratified analysis according to the age at which ownership began. This internet survey-based study aimed to resolve the limitations of the small sample size and restricted age range of subjects in our previous study.

## Materials and methods

### Participants

We used data collected in a survey conducted by the Japan Pet Food Association in 2021 [16]. Briefly, the Japan Pet Food Association has conducted annual internet surveys since 2004 to determine the number of animals in each household and the conditions of their living environment. In October 2021, the Japan Pet Food Association commissioned an internet survey to a research company, which surveyed individuals aged 20 to 79 years who confirmed their willingness to participate. The internet survey included 2030 dog and/or cat owners and 2287 non-dog or cat owners.

Informed consent was obtained on the web-site before an internet survey. All participants provided informed consent, and all protocols were approved by the Ethics Committee at the National Institute for Environmental Studies. We adhered strictly to the Declaration of Helsinki. Authors could not access to information that could identify individual participants during or after data collection.

### Definition of dog and cat ownership

Participants were retrospectively asked if they had owned a dog or cat. Those who reported that they had owned a dog or cat were asked to indicate the age at which ownership started and ended. If the participants had owned pets at various stages of life, they were asked to indicate the age ranges for a maximum of five periods of ownership. We extracted the earliest age at which pet ownership began for each participant.

### Definition of asthma and allergy

We defined participants with asthma as those who has been clinically diagnosed with asthma and allergy. Participants were retrospectively asked whether a physician had diagnosed them with asthma and allergies (yes or no) [17, 18]. If the participant answered yes to either condition, they were asked to indicate the age at which the condition started and ended. If they had been diagnosed with the condition at various stages of life, they were asked to indicate the age ranges for a maximum of 2 periods during which they had the condition. We extracted the earliest age of asthma onset for each participant.

### Other measurements

We used data on sociodemographic characteristics, including sex, age, type of residence (owned house, owned apartment, rental house, rental apartment, company house, and other), household income (0 to 2 million yen, 2 to 4 million yen, 4 to 6 million yen, 6 to 8 million yen, 8 to 10 million yen, 10 to 12 million yen, 12 to 15 million yen, 15 to 20 million yen, ≥20 million yen, unknown, and refused to answer), number of family members, and area of residence (Hokkaido, Tohoku, Kanto, Keihin, Hokuriku, Tokai, Hanshin, Chugoku, Shikoku, and Kyushu) at the time of the survey.

The follow-up period was defined as the period from the earliest age at which the participant was exposed to a dog and/or cat through ownership to the age at which outcomes of asthma were identified. For participants who had not owned a dog and/or cat, the follow-up period was calculated from age 0. Furthermore, for participants who had never developed asthma, the follow-up period ended at the time of the survey.

### Statistical analyses

Because we wanted to focus on dog and/or cat ownership before onset of asthma, we treated all participants who owned a dog and/or cat after developing asthma as having had no exposure. Moreover, we excluded participants whose earliest age of exposure to a dog or cat was the same as that at which they developed asthma. Of the 4317 participants (2030 dog and/or cat owners and 2287 non-dog or cat owners) in the 2021 survey, we excluded 27 and 9 subjects whose earliest age of exposure to a dog and cat, respectively, was the same as that at which they developed asthma. Thus, we received valid data from 4290 participants for analysis of dog ownership and 4308 participants for analysis of cat ownership.

In the analysis, we first compared sociodemographic characteristics among dog and/or cat owners and non-pet owners using a two-tailed Pearson’s chi-squared or t-test [19]. Second, we examined the association of dog and/or cat ownership with asthma onset using a two-tailed Pearson’s chi-squared test [19]. Third, independent associations of dog and/or cat ownership with the onset of asthma were examined using binomial logistic regression analysis after adjustment for sex, type of residence, household income, number of family members, medical history of allergy, and follow-up period [20]. We additionally confirmed independent associations of dog and/or cat ownership with the onset of asthma in a mixed effects model with area of residence as a random effect [21]. Finally, stratified analysis was conducted according to the earliest age at which participants were exposed to a dog or cat through ownership, namely up to 10, 20, 30, 40, 50, and 60 years old, using binomial logistic regression analysis. All statistical analyses were conducted using SPSS (version 23.0; IBM Corp, Armonk, NY, USA) and SAS (version 9.4; SAS Institute, Inc., Cary, NC, USA). P values of less than .05 were considered statistically significant.

## Results and discussion

The sociodemographic characteristics of dog and cat owners and non-dog or cat owners are shown in Table 1. Of the study population, 41.2% had owned a dog and 26.5% had owned a cat. Over a mean (SD) follow-up period of 48.4 (19.5) years, 5.7% of dog owners and 14.8% non-dog owners developed asthma (χ^2^ = 87.3, p<0.001). Meanwhile, over a mean (SD) follow-up period of 48.5 (19.5) years, 5.6% of cat owners and 13.5% of non-cat owners developed asthma (χ^2^ = 52.1, p<0.001).

**Table 1:** Sociodemographic characteristics of dog/cat owners and non-pet owners in Japan.

Variable	Experience owing a dog (n = 1769, 41.2%)	No experience owning a dog (n = 2521, 58.8%)	P-value	Experience owning a cat (n = 1143, 26.5%)	No experience owning a cat (n = 3165, 73.5%)	P-value
Sex (male, %)	46.7	51.2	.004	44.3	51.2	< .001
Age (years)	54.1 (14.9)	50.4 (16.4)	< .001	53.6 (15.7)	51.2 (16.0)	< .001
Type of residence (%)			< .001			.006
Owned house	65.9	52.6		62.4	56.5	
Owned apartment	12.6	14.9		12.4	14.6	
Rental house	2.9	2.9		3.2	2.8	
Rental apartment	17.1	26.9		20.1	23.8	
Company house	0.7	1.3		0.6	1.2	
Other	0.8	1.3		1.2	1.1	
Household income (%)			.001			.083
0 to 2 million yen	9.5	13.4		13.0	11.3	
2 to 4 million yen	29.3	30.3		30.2	29.9	
4 to 6 million yen	26.4	24.2		22.8	26.0	
6 to 8 million yen	14.5	15.0		14.6	14.7	
8 to 10 million yen	10.4	8.8		9.3	9.5	
10 to 12 million yen	4.1	3.5		4.4	3.6	
12 to 15 million yen	3.0	2.7		3.0	2.8	
15 to 20 million yen	1.1	1.1		1.5	1.0	
≥20 million yen	1.6	0.8		1.0	1.2	
Unknown	0	0.1		0.1	0	
Number of family members	2.7 (1.3)	2.6 (1.3)	.024	2.7 (1.3)	2.6 (1.3)	.948
Area of residence (%)			< .001			.103
Hokkaido	4.6	4.7		4.6	4.6	
Tohoku	6.6	7.5		8.3	6.7	
Kanto	7.2	6.7		8.1	6.5	
Keihin	24.9	31.4		26.4	29.6	
Hokuriku	5.4	5.9		6.6	5.3	
Tokai	11.5	10.7		11.1	11.0	
Hanshin	18.1	15.4		15.0	17.1	
Chugoku	6.4	5.3		5.8	5.8	
Shikoku	4.2	2.4		3.0	3.2	
Kyushu	11.0	10.0		10.9	10.2	
Medical history of allergy (%)	29.8	32.8	.037	30.1	32.3	.166
Onset of asthma (%)	5.7	14.8	< .001	5.6	13.5	< .001
Follow-up (years)	52.0 (17.1)	45.9 (20.7)	< .001	51.5 (17.6)	47.3 (20.1)	< .001

Data indicate % or mean (standard deviation). P-values were calculated using a two-tailed Pearson’s chi-squared test or t-test.

According to a recent study, some states in the US have established restrictions on where dogs can be kept based on the weather and outdoor temperature [22]. In Japan, it was common to keep dogs and cats outdoors until a few decades ago and, while there are currently no restrictions, it has become more common to keep pets indoors in recent years. Therefore, we hypothesized that middle-aged and older Japanese people may tend to keep dogs and cats outdoors, while younger people may keep them indoors. The proportion of dog and cat owners in Japan is 11.3% and 9.8%, respectively [16], which is lower than that in Western countries. In our study of participants from a wide range of age groups in Japan, the living environments of dogs and cats differed from those in Western countries.

Table 2 shows the independent associations of dog and cat ownership with the onset of asthma in Japanese. Results of binomial logistic regression analysis revealed that participants who had not owned a dog had an odds ratio (OR) of 2.01 (95% confidence interval (CI): 1.45–2.78) of developing asthma compared with those who had owned a dog after adjustment for sex, type of residence, household income, number of family members, medical history of allergy, and follow-up period. Among those analyzed for cat ownership, participants who had not owned a cat had an OR of 2.24 (95%CI: 1.56–3.23) of developing asthma compared with those who had owned a cat after adjustment for the same confounders. Both dog and cat ownership showed a significant association with onset of asthma in the mixed effects model, with variability observed by participants’ area of residence (P<0.01). The adjusted ORs of developing asthma were 2.01 and 2.24 among participants who had not owned a dog or cat compared to those who had, making the adjusted ORs of developing asthma 0.50 and 0.45 among dog and cat owners compared to non-dog or cat owners. The crude OR and relative risk (RR) of developing asthma was 0.35 and 0.35 for those who owned a dog and 0.40 and 0.40 for those who owned a cat, respectively, compared with non-dog or -cat owners. A meta-analysis of studies from Western countries reported an RR of asthma onset of 1.10 (95%CI: 0.96–1.26) among those with dog exposure and an RR of 0.72 (95%CI: 0.55–0.93) among those with cat exposure from 5 cohort studies [13]. The association of RR of asthma onset with dog ownership reported in the meta-analysis differs from that in the present study. Previous study in Japan reported that dog and cat ownership from toddler-age does not increase the risks of wheezing and asthma compared with never owners [14]. The present retrospective study of participants from a wide range of age groups in Japan, where the living environment of dogs and cats differ from that in Western countries, largely supports accumulated evidence in Japan and suggests that dogs and cats are equally effective at preventing asthma.

**Table 2:** Independent associations of dog and/or cat ownership with the onset of asthma in Japanese from birth to old age.

	All participants: Adjusted OR (95%CI)	Stratified analysis according to the earliest age at which participants owned a pet					
		Up to 10 years old: Adjusted OR (95%CI)	Up to 20 years old: Adjusted OR (95%CI)	Up to 30 years old: Adjusted OR (95%CI)	Up to 40 years old: Adjusted OR (95%CI)	Up to 50 years old: Adjusted OR (95%CI)	Up to 60 years old: Adjusted OR (95%CI)
Owned a dog							
Yes†	1	1	1	1	1	1	1
No	2.01 (1.45–2.78) **	2.15 (1.41–3.27) **	2.19 (1.52–3.14) **	2.14 (1.52–3.00) **	2.10 (1.51–2.93) **	2.03 (1.47–2.82) **	2.01 (1.45–2.78) **
Owned a cat							
Yes†	1	1	1	1	1	1	1
No	2.24 (1.56–3.23) **	2.02 (1.21–3.34) **	2.31 (1.50–3.54) **	2.44 (1.64–3.63) **	2.35 (1.61–3.43) **	2.27 (1.57–3.28) **	2.25 (1.56–3.24) **

OR = odds ratio; CI = confidence interval

† Reference group

* p < .05

** p < .01.

Adjusted for sex, type of residence, household income, number of family members, medical history of allergy, and follow-up period.

Table 2 also shows the results of stratified analysis of associations between dog and cat ownership and the onset of asthma. Regarding dog ownership, participants who did not own a dog until age 40 years had an OR of asthma onset of 2.10 (95%CI: 1.51–2.93) compared to those who did, and those who did not own a dog when younger had higher ORs of developing asthma, suggesting that a critical window during which dog exposure may prevent asthma is early life in Japan. For cat ownership, although the OR of asthma onset among participants who did not own a cat until age 10 years was relatively low, the OR related to owning a cat was similar across all age categories compared with not owning a cat. These results suggest that the protective effect of cat exposure on the onset of asthma may be constant across all ages in Japan.

Although a previous study also suggested that owning dogs and cats protects against the development of allergies [15], the precise mechanism underlying the link between dog and/or cat exposure and onset of asthma is unclear. We propose two possible explanations. First, exposure to pets in the household, such as living with furry pets, is associated with a different microbiome profile in early life [23, 24], and previous studies have reported that living with a pet is related to microbial alterations [25–30]. These results support the biodiversity hypothesis, which states that contact with natural environments enriches the human microbiome, promotes immune balance and protects from allergic and inflammatory disorders [31]. Thus, the composition of the human gut microbiome induced by living with dogs and cats might play a key role in preventing asthma. Second, pet ownership is strongly associated with indoor levels of bacterial endotoxins [32], which protect against allergies by inducing the production of A20 in lung epithelial cells [33]. Thus, A20 expression induced by exposure to dogs and cats might negatively regulate multiple inflammatory signaling pathways. We speculate that these potential mechanisms of the association of dog and cat ownership with asthma may come into play in young people who own dogs, and people of all ages who own cats.

Among the 4290 participants included for analysis of dog ownership at the time of the survey, 49.4% were male and the mean (SD) age was 51.9 (15.9) years. Regarding type of residence, 58.1% lived in a house they owned, 22.8% lived in a rental apartment, and 14.0% lived in an apartment they owned. Median household income was 4 to 5 million yen and 55.9% of participants earned under 5 million yen. Mean (SD) number of family members was 2.7 (1.3). Regarding area of residence, 28.7% of participants lived in the Keihin area, 16.5% in the Hanshin area, and 11.0% in the Tokai area. Further, 31.6% had a medical history of allergy and 11.0% experienced onset of asthma. Household income, number of family members, and prevalence of asthma of participants in this study largely overlapped with those of a comprehensive survey of living conditions [34], the national census in Japan [35], and the nationwide study [36].

The main strength of this study is that we collected data from Japanese people who themselves, together with their pets, live in a very different environment from that of Western countries. This study is the first to show an association of dog and cat ownership with the onset of asthma in Japan. Second, analysis stratified by the age at which participants first became exposed to pets allowed us to identify any critical windows during which dog and cat ownership protects against asthma development. This study is the first to identify critical windows of dog and cat ownership that have an impact on asthma.

Notwithstanding these strengths, the study also had some limitations. First, the data were collected through an internet survey. Although participants varied widely by age and distribution throughout Japan, a degree of selection bias related to participants’ ability to respond to an internet survey may have been present. Second, due to the nature of the survey, a greater proportion of subjects in this study owned dogs and cats than overall in Japan [16]. Although a larger proportion with pet exposure is unlikely to affect the association with asthma onset, some potential bias might still be present. Third, although the variables used in this study were the same as those inquired about in the survey, the type of residence, household income and number of family members—which were used as covariates in the binomial logistic regression analysis—were not assessed at the time during which participants first owned a dog and/or cat. Thus, a degree of recall bias related to these variables may be present. Future studies that control for these and other potential biases are necessary to confirm the protective effect of dog and cat exposure on the onset of asthma in Japanese populations.

## Conclusions

This retrospective study is the first to show that dog and cat ownership is associated with a protective effect on the onset of asthma in Japanese. Adjusted ORs of asthma onset were 2.01 and 2.24 among those who had not owned a dog and cat, respectively, compared to those who had, after controlling for sociodemographic characteristics. Stratified analysis by age at which participants were first exposed to dogs and cats through ownership suggests that the critical window during which dog exposure can prevent the development of asthma is early life whereas the protective effect of cat exposure is constant across all ages in Japan.
